# TSH adenoma and syndrome of resistance to thyroid hormones—Two cases report of syndrome of inappropriate secretion of thyrotropin

**DOI:** 10.1002/brb3.2081

**Published:** 2021-03-10

**Authors:** Fang Deng, Ze‐yu Yang, Yu‐ping Zhang, Yu‐lin Wang, Jiong‐yu Hu, Fan Zhang

**Affiliations:** ^1^ Department of Endocrinology Southwest Hospital Third Military Medical University (Army Medical University) Chongqing China; ^2^ Breast and Thyroid Surgical Department Chongqing General Hospital University of Chinese Academy of Sciences Chongqing China

**Keywords:** resistance to thyroid hormones, syndrome of inappropriate secretion of thyrotropin, TSH adenoma

## Abstract

SITSH (syndrome of inappropriate secretion of thyrotropin) is a rare clinical state defined as uninhibited serum thyroid stimulating hormone in the presence of elevated thyroid hormone. This state is complicated and mainly caused by the abnormal feedback of hypothalamus–pituitary thyroid axis. The TSH adenoma (TSH‐oma) and resistance to thyroid hormones (RTH) are the main etiologies of SITSH. As is well known that the treatment strategies of RTH and TSH‐oma are apparently different, thus identifying the difference between RTH and TSH‐oma is of great significance for the diagnosis and treatment of SITSH.

**Case description:**

A 62‐year‐old man with a state of elevated thyroid hormones and inappropriate elevated serum TSH level was hospitalized in 2016. Results of the pituitary enhanced magnetic resonance imaging and the somatostatin test respectively demonstrated a space‐occupying lesion of pituitary and an elevated serum sex hormone binding globulin (SHBG) and inhibited TSH secretion, which indicated the occurrence of TSH‐oma. In 2019, a 23‐year‐old girl with a state of elevated thyroid hormones and inappropriate normal serum TSH was hospitalized. Interestingly, whole exome sequencing detection suggested a pathogenic mutation in thyroid hormone receptor β (*THRB*) gene, which has been shown to be associated with RTH.

**Conclusions:**

The difference between TSH‐oma and RTH ought to be clarified for their accurate diagnose and treatment. The clinical experiences of the two cases reported here suggest that more detail information such as family medical history, serum SHBG level, and THRB gene test is helpful for the diagnose and treatment of TSH‐oma and RTH. Additionally, we also summarized the identification points, diagnosis process, and treatment strategies for these two rare diseases.

## Graphical abstract

Due to the complicated manifestation of TSH‐oma and RTH, the diagnosis and treatment often delayed. The clinical experiences of the TSH‐oma and RTH suggest that more information should be identified. In this article, by comparing TSH‐oma and RTH, we summarized the identification points, diagnosis process and treatment strategies of these two rare diseases.

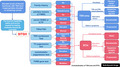

## 1 INTRODUCTION

SITSH is a clinical state defined as inappropriately uninhibited serum thyrotropin (TSH) in the presence of elevated thyroid hormones (Han et al., 2020). TSH adenoma (TSH‐oma) and resistance to thyroid hormones (RTH) are the two main etiologies of SITSH. The mobility of TSH‐oma is around 1 case per million, which accounts for 0.5% to 3% of all pituitary tumors (Amlashi & Tritos, 2016; Beck‐Peccoz et al., 2000). TSH‐oma is the main cause of central thyroid dysfunction, which could interpret the corresponding changes of pituitary mass and thyrotoxicosis (Amlashi & Tritos, 2016; Beck‐Peccoz et al., 2000). Due to the complicated manifestation and limited clinicians' understanding, the diagnosis and treatment of TSH‐oma are often delayed (Cossu et al., 2019; Fujio & Yoshimoto, 2018).

RTH is characterized by hyposensitivity to thyroid hormone of corresponding targeting tissues. Almost 85% patients with RTH have the mutation of thyroid hormone receptor α (*THRB*) gene (Cossu et al., 2019). The mutation of thyroid hormone receptors (THR) decreases their affinity for thyroid hormone and may result in an inhibitory effect on normal thyroid hormone receptor. According to the resistance degree of different tissues to thyroid hormone, RTH could be divided into 3 types: generalized resistance, selective pituitary resistance, and selective peripheral resistance. Most patients with generalized resistance to thyroid hormone have few manifestations, and some of them may have hypothyroidism. Selective pituitary resistance usually occurs in adults with the main clinical feature of thyrotoxicosis. Selective peripheral resistance is extremely rare. The clinical features are characterized by enlarged goiter, elevated serum TH level, and hypothyroidism (Kassak et al., 2017; Murata, 2012).

## 2 CASE REPORT

### 2.1 Case one

**FIGURE 1:**
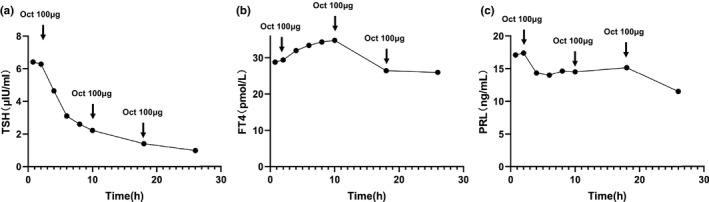
Somatostatin suppression test of case one (100µg Octreotide subcutaneous injection per 8 hr). (a) TSH changes before and after Octreotide injection. (b) FT4 changes before and after Octreotide injection. (c) PRL changes before and after Octreotide injection. Blood samples were obtained before and 2, 4, 6, 8, 16 and 24 hr after the first injection of 100µg Octreotide. TSH, Thyrotropin; FT4, Free serum thyroxine; PRL, prolactin; Oct, Octreotide

**FIGURE 2:**
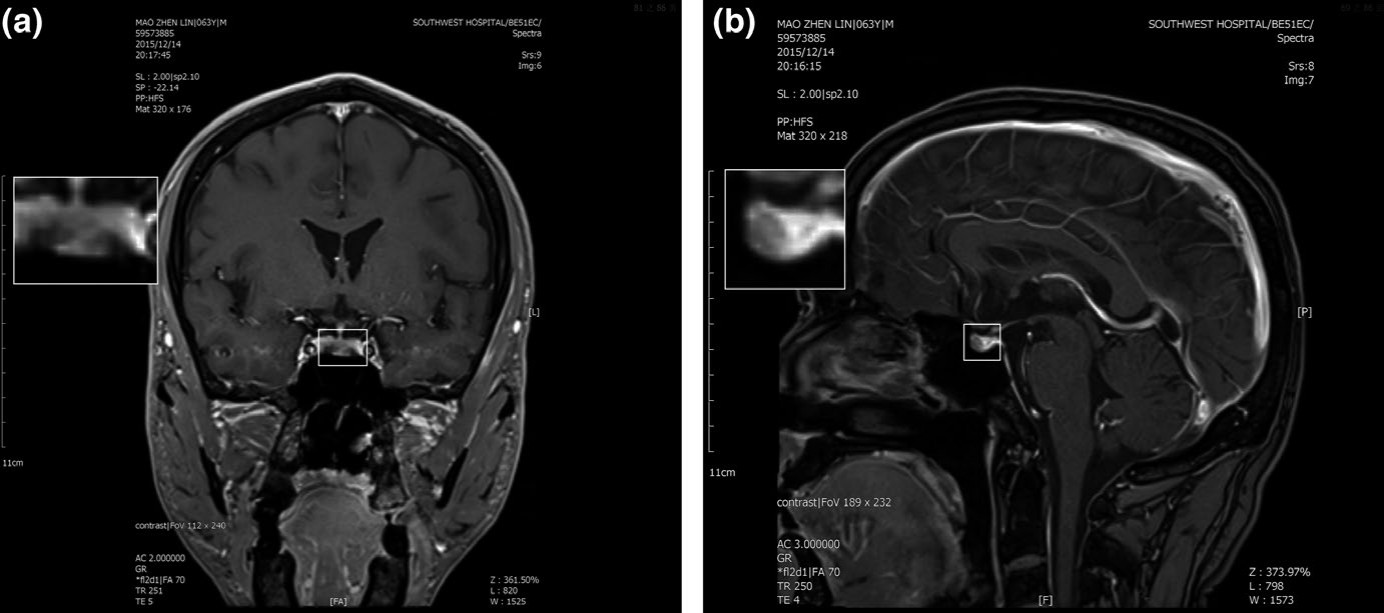
Pituitary enhanced MRI scan of case one showed a small round low signal shadow

A 62‐year‐old man was referred to our hospital due to thyrotoxicosis. At first sight, he complained of changes in stool traits for 11 months and his hands shook and palpitation for more than 2 months in January 2016. His past history and family history were unremarkable. He was 173 cm tall, weighed 63 Kg, and the BMI (body mass index) was 21.5 kg/m^2^. The pulse rate was 72 beats/min, blood pressure was 120/82 mmHg, and body temperature was 36.5 ℃. Routine blood tests, including complete blood cell count, electrolyte, and renal and liver function were all within normal limits. Thyroid function test was performed and revealed a state of elevated thyroid hormones with an inappropriate increasing of serum TSH level (Table 1). No obvious abnormality in the cortisol axis and gonad axis was found. Additionally, an elevated serum sex hormone binding globulin (SHBG) (84.9 nmol/L; normal range: 13–71 nmol/L) was revealed through serum hormone test. Results of the somatostatin suppression test (Han et al., 2020) demonstrated a significant serum TSH inhibition (Figure 1). Cardiac ultrasound revealed a mild regurgitation of the mitral, tricuspid, aortic, and pulmonary valves, along with a decreased early diastolic function of the left ventricle (Left ventricular ejection fraction, EF = 59%; fractional shortening, FS = 32%). Thyroid radionuclide imaging and iodine uptake rate examination showed no remarkable changes in thyroids. Pituitary enhanced magnetic resonance imaging (MRI) demonstrated a small round low signal shadow in pituitary region which indicated a pituitary microadenoma (Figure 2). Moreover, gene tests were performed and no *THRB* mutation was found. The diagnosis of TSH‐oma was made based on the above‐mentioned clinic performances and associated tests. For the pathological diagnosis and further treatment, the biopsy and surgery resection of pituitary microadenoma were recommended but were rejected for personal reasons. Then, somatostatin therapy was not administered for the high costs and long‐term medication. Therefore, this patient underwent routine clinical follow‐up. So far, the patient still suffers from hands shaking and palpitation. Recent cardiac ultrasound showed reduced left ventricular ejection function (EF = 53%, FS = 27%). Defecation and urination are basically normal, and no significant change in body weight. Thyroid function test showed significant elevation of serum FT3, FT4, and TSH, the degree of which were not significantly changed compared with before.

**TABLE 1:** Endocrine hormonal test results

Indicator	Reference value	Case one	Case two
General			
TT4 (nmol/L)	66–181	193.7	149.9
FT4 (pmol/L)	12–22	35.97	23.13
TT3 (nmol/L)	1.3–3.1	3.68	2.36
FT3 (pmol/L)	3.1–6.8	7.58	6.96
TSH (uIU/ml)	0.27–4.20	5.75	1.2
ACTH (pg/ml)	5–60	46.10	25.61
GH (ng/mL)	0.55–4.74	0.03	0.19
Cortisol rhythm test (nmol/L)			
08:00	181.83–716.3	692.41	704.09
16:00		463.35	284.98
24:00			281.41
08:00 (Next day)		624.08	589.83
Sex hormones	(Male/Female)		
E2 (pg/mL)	20−75/24−114	33.00	39.00
P (ng/mL)	0.1–0.84/0.31–1.52	0.61	0.31
FSH (nmol/L)	1.27–19.26/3.85–8.78	16.52	0.82
LH (pmol/L)	1.24–8.62/2.12–10.89	7.77	0.15
PRL (uIU/ml)	2.64–13.13/3.34–26.72	9.61	8.2
T (ng/mL)	1.75–7.81/0.1–0.75	4.81	0.12

Note

The reference ranges of E2, P, FSH, LH, and PRL are shown in follicular phase of women.

Abbreviations: ACTH, adrenocorticotropic hormone; E2, Estradiol; FSH, follicle stimulating‐hormone; FT3, free triiodothyronine; FT4, free thyroxine; GnRH, gonadotropin‐releasing hormone; LH, luteinizing hormone; P, progesterone; PRL, prolactin; T, testosterone; TSH, thyrotropin.

### 2.2 Case two

**FIGURE 3:**
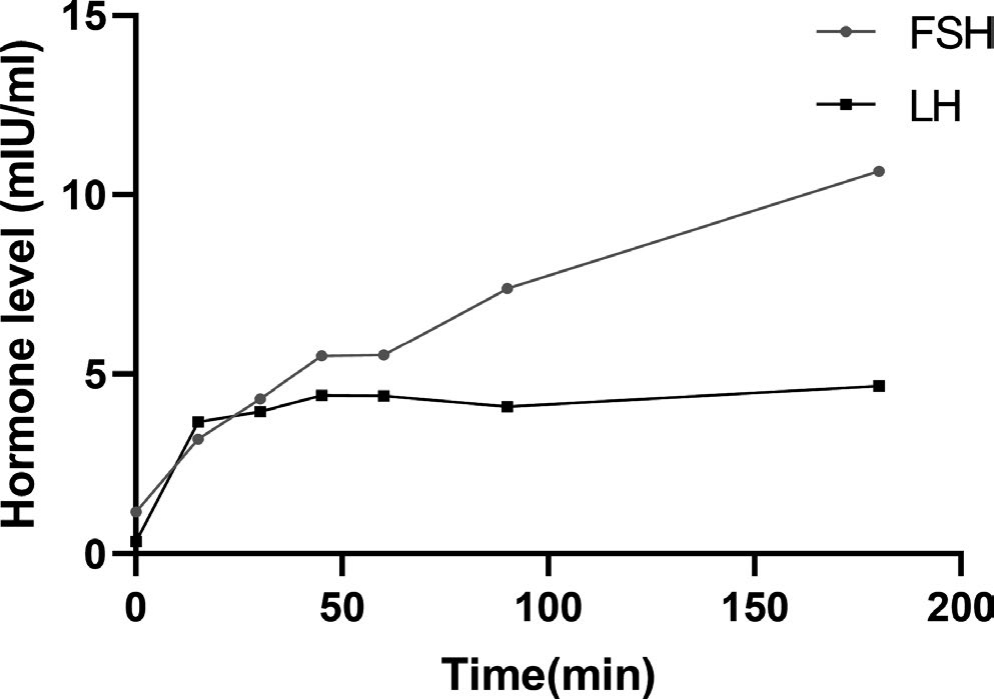
GnRH excitement test of case two. Blood samples were obtained before and 15, 30, 45, 60, 90 and 180 min after injecting 100µg GnRH. FSH, follitropin; LH, luteinizing hormone

**FIGURE 4:**
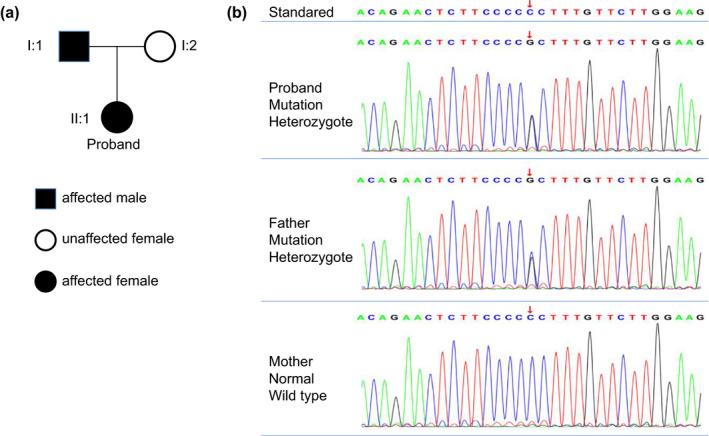
*THRB* mutation of case two. (a) The pedigree of the family with *THRB* mutation is shown. (b) Partial sequences of exon of *THRB* gene and the mutation site. The patient and her father have a heterozygous mutation (Chromosome site: Chr 3:24 164404; exon site: 12 c.1357 C > G) denoted by the arrow

A 23‐year‐old girl was referred to our hospital in March 2019 due to a 5‐year menstrual disorder with pulse 120 beats/min. Blood pressure was 113/85 mmHg and body temperature was 36.6℃. Her past history was unremarkable. As to her family medical history, her father was diagnosed with “Abnormal thyroid function” about. She was 164 cm tall, weighed 52 kg, and the BMI was 19.33 kg/m^2^. Routine blood tests, including blood cell count, renal and liver function, and electrolyte were all within normal limits. Serum hormone testing indicated that the patient had a state of elevated thyroid hormones with an inappropriate normal serum TSH level, along with reduced Follicle‐stimulating hormone (FSH) and Luteinizing hormone (LH) (Table 1). The gonadotropin‐releasing hormone (GnRH) excitement test showed significant increasing of both LH and FSH (Figure 3), providing evidence for the diagnosis of hypothalamic amenorrhea, while the SHBG was in the normal range (42.60 nmol/L; normal range: 13–71 nmol/L). In addition, pituitary enhanced MRI, ultrasound, thyroid radionuclide imaging, and iodine uptake rate examination showed no remarkable changes. Gene tests indicated that the patient and her father had a pathogenic heterozygous mutation of *THRB* gene (Chromosome site: Chr 3:24 164,404; exon site: 12 c.1357 C > G) (Figure 4). Based on this data, the patient was diagnosed with hypothalamic amenorrhea and RTH. Then, the GnRH pulse pump was used to maintain the secretion of sex hormones. TRIAC (triodothyroacetic acid) is the prioritized recommendations for RTH treatment (Murata, 2012), but it was difficult to obtain. Otherwise, according to the close observation during hospitalization, there were no symptoms related to thyrotoxicosis or hypothyroidism such as panic, palpitations, and sweating were found. Thus, clinical follow‐up is recommended as a priority and symptomatic treatment should be carried out if needed. After one‐year follow‐up, the symptoms of hypothalamic amenorrhea were gradually subsided, the menstrual cycle gradually became normal and the secretion of related sex hormones was elevated with little changes of RTH.

## 3 DISCUSSION

**FIGURE 5:**
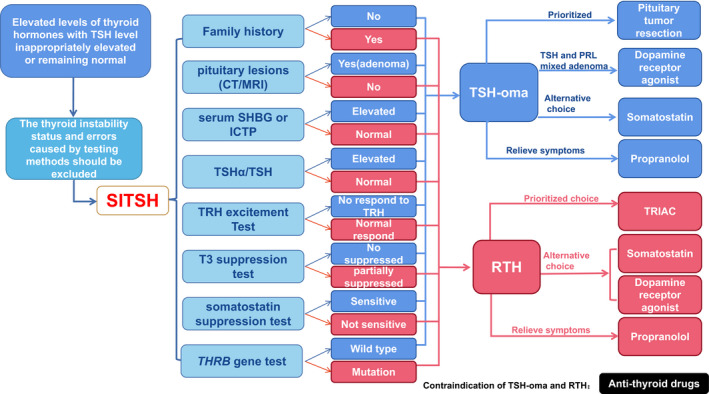
Diagnosis and treatment flow chart of SITSH

The basic feature of SITSH is that serum thyroid hormones increase with the TSH level inappropriately elevated or remaining normal (Murata, 2012). It is currently believed that SITSH is mainly caused by RTH or TSH‐oma. Failure to accurately diagnose may result in inappropriate treatment. In order to better understand RTH and TSH‐oma, we summarized the predominant features of these two cases as shown in Table 2 and carried out several diagnosis and treatment procedures according to 2013 European Thyroid Association Guidelines for diagnosis and treatment of TSH‐oma (Figure 5).

**TABLE 2:** differential diagnosis and treatment features of TSH‐oma and RTH

	TSH‐oma (case one)	RTH (case two)
Diagnosis		
Family history	No	Yes
Imageology test of pituitary	Adenoma‐like mass	Normal
Serum SHBG	Elevated	Normal
THRB gene test	Normal	Mutation
Treatment		
Pituitary tumor resection	Preferred treatment	Not suggested
TRIAC	Not suggested	Prioritized choice
Somatostatin	Alternative choice	Alternative choice
Dopamine receptor agonist	Used in TSH and PRL mixed adenoma	Alternative choice
Propranolol	Relieve symptoms	Relieve symptoms
Anti‐thyroid drugs	Contraindication	Contraindications

The TSH adenoma is the most rare form of functional pituitary adenoma (Tjornstrand & Nystrom, 2017). The clinical manifestation of functional TSH‐oma varies from disparate age groups. Typically, the mainly clinical manifestation of TSH‐oma is thyrotoxicosis, which is associated with the abnormal levels of thyroid hormones and TSH.TSH‐oma is often considered as SITSH (Beck‐Peccoz et al., 2019; Fujio & Yoshimoto, 2018). Patients with TSH‐oma usually have three kinds of manifestations: first, thyroid‐related manifestations including thyrotoxicosis and goiter (Beck‐Peccoz et al., 2019); second, manifestations of pituitary mass including visual field defect, headache etc (Beck‐Peccoz et al., 2019; Hwang et al., 2020); third, the manifestations of abnormal pituitary secretion including lactation, acromegaly, menstrual disorders, sexual dysfunction etc (Beck‐Peccoz et al., 2000; Elsarrag et al., 2020). Diagnosis of TSH‐oma depends on its unique TSH secretion feature and a confirmed adenoma‐like mass through imageology tests. In this case, the laboratory findings indicated the elevated levels of serum free T3 and free T4 along with the increased serum TSH. Visual confirmation of a microadenoma‐like mass was found with MRI. According to Rulai Han et,al., the TSH inhibition rate greater than 44.46% in somatostatin suppression test was suggested as an important diagnostic criterion of TSH‐oma (Beck‐Peccoz et al., 2000; Elsarrag et al., 2020). In this case, we found and the serum TSH decreased by more than 84% in the somatostatin suppression test (Figure 1a), which is consistent with the features of TSH‐oma. After the diagnosis of TSH‐oma, surgery is the prioritized treatment strategy.

As is firstly introduced by Refetof in 1967, RTH is defined as a rare autosomal dominant or recessive genetic disease (Refetoff et al., 1967). Due to the heterogeneity of gene mutation, clinical manifestations may be heterogeneous (Smallridge et al., 1989). Patients with RTH have various clinical manifestations, such as goiter, thyroid nodules, tachycardia, mental retardation, attention deficit hyperactivity disorder, color blindness, short stature, reduced bone mass, repeated ear and throat infections, hearing impairment, and even damage to the central nervous system (Rivas & Lado‐Abeal, 2016; Smallridge et al., 1989).

At present, the pathogenesis of RTH is not fully understood, and the mutations in THR genes are regarded as the most important etiology for RTH. Previous studies have shown that there are two thyroid hormone receptor genes in mammalian cells, the thyroid hormone receptor β (*THRB*) gene on chromosome 3, and the thyroid hormone receptor α (*THRA*) gene on chromosome 17 (Persani & Campi, 2019;; Rurale et al., 2019). Through alternative splicing of the two genes, it can be expressed as 4 functional subtypes: THα1, THβ1, THβ2, and THβ3 (Cheng, 2000; Rurale et al., 2019). These four different subtypes distribute in different tissues. THRα1 is mainly expressed in the gastrointestinal tract, heart, skeleton, muscle, and central nervous system, and lowly expressed in kidney, skeletal muscle, lung, heart, and liver. THRβ1 is mainly distributed in brain, liver, kidney, heart, and thyroid. THRβ2 is mainly distributed in hypothalamus, pituitary, cochlea, and retina. THRβ3 is mainly distributed in kidney, liver, and lung (Cheng, 2000; Kohrle, 2018; Samarut & Plate roti, 2018). RTH patients could show normal, or insensitive to thyroxine of the whole body or some specific tissues, or organs (Smallridge et al., 1989). The significant difference in clinical symptoms may be related to the different distribution of thyroid hormone receptor in different tissues (Toumba et al., 2019). The location, degree of mutated gene expression, and the effectiveness of hormone compensation jointly determine the clinical manifestations of patients (Vela et al., 2019). As shown in Table 2, the use of TRIAC, somatostatin and dopamine receptor agonists are the main treatment strategies, while the need for treatment depends on the patient's requirements and the clinical manifestations.

For the second case, the *THRB* gene mutation of the second case (Chromosome site: Chr 3:24 164404; exon site: 12 c.1357 C > G) had typical RTH manifestations without any significant performance of thyrotoxicosis or hypothyroidism. THRβ is mainly distributed on the pituitary gland, liver, and kidney. *THRB* mutations in the pituitary gland could prevent TSH from being inhibited by elevated thyroid hormones, which is believed to be the reason for the SITSH. Similarly, the mutation of *THRB* in liver cells could reduce the sensitivity of liver tissue to thyroid hormones, and this may be the reason why SHBG did not increase as the thyroid hormone raised (Mok et al., 2013; Murata, 2012). Moreover, the increased heart rate of this patient could be induced by THRα1 expression in myocardial cells (Mok et al., 2013; Murata, 2012). It is common for the reported cases of RTH without obvious clinical manifestations like this patient y (Murata, 2012). In fact, the diagnoses for these patients remain complicated, and more critically, the confirmed diagnosis mainly depends on the gene sequencing testing. So, we need to strengthen our understanding of this disease for better diagnosis and treatment.

In addition to the diagnosis of RTH, this patient also has hypothalamic amenorrhea. Is there a potential connection between RTH and hypothalamic amenorrhea? By consulting the previous literature, none clear correlation between RTH and hypothalamic amenorrhea was reported to our knowledge. Thus, we suppose that the mutation of *THRB* in hypothalamic region (especially those cells related to gonadotropin‐releasing hormone secretion) could result in the dysfunction of gonadotropin releasing. However, this hypothesis needs to be confirmed by follow‐up experiments and clinical investigations in future.

## 4 CONCLUSION

The cases reported here presented our experience in the diagnosis and management of RTH and TSH‐oma. The clinical experiences of the two cases reported here suggest that more detail information such as family medical history, serum SHBG level, and THRB gene test is helpful for the diagnose and treatment of TSH‐oma and RTH. In addition, we summarized the identification points, diagnosis process, and treatment strategies of these two rare diseases.

## CONFLICTS OF INTEREST

The authors declare that there is no conflict of interest of this paper.

## AUTHOR CONTRIBUTION

Fan Zhang and Jiong‐yu Hu contributed to the conception and manuscript revision of the study; Fang Deng performed the data collection; Fang Deng and Zeyu Yang contributed significantly to analysis and manuscript preparation; Zeyu Yang performed the data analyses and wrote the manuscript; Yu‐ping Zhang, Yu‐lin Wang helped perform the analysis with constructive discussions.

## ETHICAL APPROVAL

According to the institution guideline, case report does not require ethical approval.

### PEER REVIEW

The peer review history for this article is available at https://publons.com/publon/10.1002/brb3.2081.

[Correction added on March 20, 2021, after first online publication: Peer review history statement has been added.]

## Data Availability

The data that support the findings of this study are available from the corresponding author upon reasonable request.
